# Envisioning Insight-Driven Learning Based on Thick Data Analytics With Focus on Healthcare

**DOI:** 10.1109/ACCESS.2020.2995763

**Published:** 2020-06-01

**Authors:** Jinan Fiaidhi

**Affiliations:** Department of Computer ScienceLakehead University 7890 Thunder BayONP7B 5E1Canada

**Keywords:** Patient insights, thick data, learning patient preferences, graph based algorithms, graph-based machine learning, graph-based transfer learning, Neo4j, twitter conversation communities

## Abstract

Detecting and analyzing patient insights from social media enables healthcare givers to better understand what patients want and also to identify their pain points. Healthcare institutions cannot neglect the need to monitor and analyze popular social media outlets such as Twitter and Facebook. To have a study success, a healthcare giver needs to be able to engage with their patients and adapt to their preferences effectively. However, data-driven decision-making is no longer enough, as the best-in-class organizations struggle to realize tangible benefits from their data-driven analytics investments. Relying on simplistic textual analytics that use big data technologies to learn consumer/patient insights is no longer sufficient as most of these analytics utilize sort of bag-of-words counting algorithms. The majority of projects utilizing big data analytics have failed due to the obsession with metrics at the expense of capturing the customer’s perspective data, as well as the failure in turning consumer insights into actions. Most of the consumer insights can be captured with qualitative research methods that work with small, even statistically insignificant, sample sizes. Employing qualitative analytics provide some kind of actionable intelligence which acquires understanding to broad questions about the consumer needs in tandem with analytical power. Generating insight, on one hand, requires sound techniques to measure consumers’ engagement more precisely and offers depth analytics to the consumer data story. On the other hand, turning relevant insights into actions requires incorporating actionable intelligence across the business by verify hypotheses based on qualitative findings by using web analytics to see if these axioms apply to a large number of customers. The first component of our visionary approach is dedicated to identifying the relationships between constituents of the healthcare pain points as echoed by the social media conversation in terms of sociographic network where the elements composing these conversations are described as nodes and their interactions as links. In this part, conversation groups of nodes that are heavily connected will be identified representing what we call conversation communities. By identifying these conversation communities several consumer hidden insights can be inferred from using techniques such as visualizing conversation graphs relevant to given pain point, conversation learning from question answering, conversations summaries, conversation timelines, conversation anomalies and other conversation pattern learning techniques. These techniques will identify and learn the patient insights without forgetting from the context of conversation communities, are tagged as “thick data analytics”. Additionally machine learning methods can be used as assistive techniques to learn from the identified thick data and build models around identified thick data. With the use of transfer learning we also can fine tune these models with the arrival of new conversations. The author is currently experimenting with these seven insights driven learning methods described in this paper with massive geo-located Twitter data to infer the quality of care related to the current COVID-19 outbreak.

## I. Introduction

The traditional solution to understanding patient experience and insights from social media is the use of web scrapers to collect patient’s feedback from notable blogs (e.g. from PatientsLikeMe.org, Drugs.com) and eliciting patient complaints from governmental systems like the FDA FAERS [1]. However, a more trendy approach is to use Big Data analytics for learning patients’ insights especially from social media [2]. Both techniques have been intensively used in the past decade with limited success to capture patient experience and explain why patients do what they do [3]. Such approaches prove to be daunting if it is built on prediction models that are based on quantitative datasets and methods which do not incorporate important consumer values and insights. According to Pink *et al.* [4] “ Big data by nature strips away context and cannot bring to light the qualitative and ethnographic nature embedded in the consumer narrative feedback to uncover people’s emotions, stories, and models of their world.” Without it, companies are basing important business decisions off incomplete data — like what led to Nokia failure in 2009 [5]. To bring context and then consumer insights is the use of qualitative research based on small samples and corpuses (“small data”). It is argued that the unique value of such qualitative research lies in data thickness. This is achieved through a process that is called thickening or thick data analytics [6]. However, the thick data research did not provide enough guides into how to mine healthcare thick data patterns from social media. In this research presenting three strategies to thicken health related narrative data extracted from social media like twitter by developing a qualitative exploration methodology that utilizes conversations’ structures, identification of related components in conversations and learning from conversations. The qualitative exploratory method seeks to define and interpret unclear phenomena through non-numerical methods of measurement that focus on meaning and insight.

## II. Healthcare-Based Insight-Driven Learning

**FIGURE 1.**
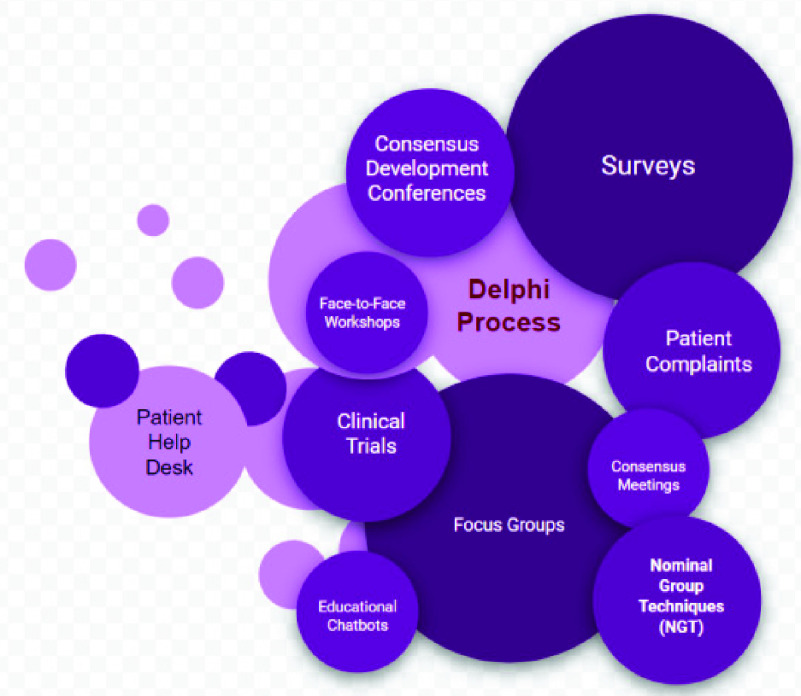
Current patient insights learning methods.

Insight-driven learning (IDL) for healthcare is all about finding methods that caregiver use for patient-centered outcomes prediction. Usually such methods incorporate patient, public and caregiver views into the process of forming predictions. However, the current methods used in healthcare involve direct patient involvement as an integral and transformative part of the methods processes. Figure 1 lists some of the notable IDL methods.

Based on these methods, patient involvement was not impactful because these methods was not successful in recruiting and retaining participants, and there were many no validation feedback from the public about the issues raised by these participatory methods. However, the Delphi method shows more promising support across the health sector as it has been recognized as an optimal method for consensus building, with use of anonymous feedback from an expert panel and statistical analysis techniques to interpret the data. The iterative nature of the Delphi process avoids some of the pitfalls of other IDL methods, such as the effects of dominant persons or the tendency to conform to a particular viewpoint [7]. Despite this promising support, Delphi process suffers from the following weaknesses [8]:

• Does not take into account widely differing opinions or large changes in public opinions (paradigm shifts [9])
• The initiator’s point of view may dominate in the analysis
• Time-consuming
• Requires high participant motivation
• The quality of the participants affects the outcomes

**FIGURE 2.**
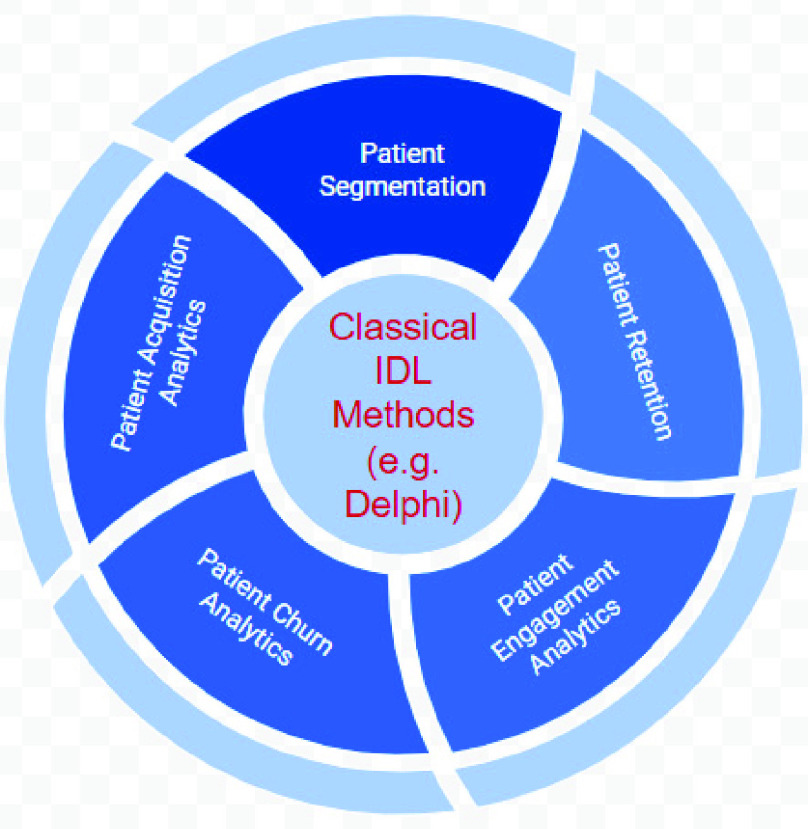
Added value techniques to enhance IDL methods.

There are more problems and concerns associated with the conceptualization and meaningful assessment and measurement of all the current IDL methods, have also been identified [10]. Each of these concerns and weaknesses points to different directions for methodological research on IDL that involve patient and public involvement with high engaging degree and empowering learning to have an impact [11]. Many healthcare institutions tried to benefit from other businesses by incorporating added value capabilities on top of the classical IDL methods to provide better predictively and higher acceptability [12]. Figure 2 illustrate these added value techniques on top of the available IDL methods.

**FIGURE 3.**
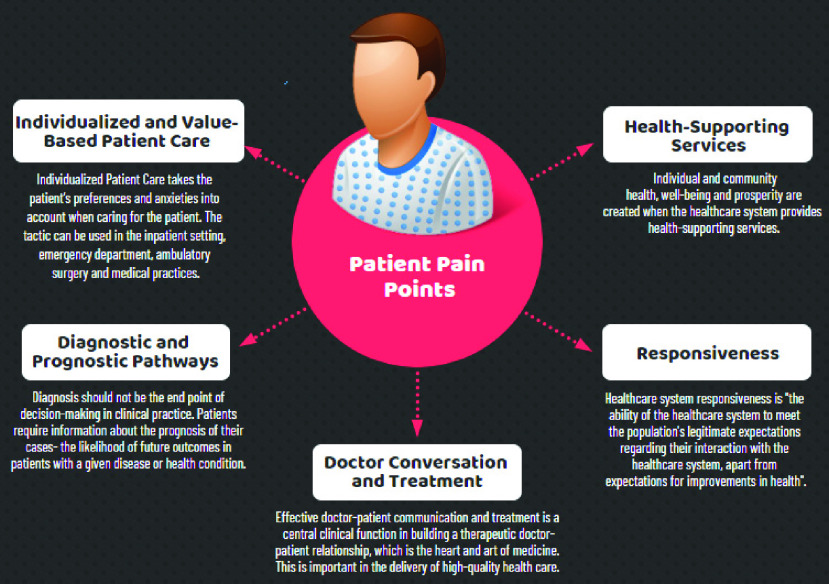
Popular patient pain points.

The added value techniques adds an extra layer on top of the analytics of the IDL methods for identifying consumer insights, however, it proved to be daunting to add more analytics on top of unsuccessful analytics. Central to having a successful IDL method is to understand the patient ‘*pain points*.’ According to a recent survey by Medici [13] reveals widespread patient frustration with the America’s healthcare system, illustrating why we need to reimagine the way we design our IDL methods if are heading to a more value-based healthcare systems [14]. IDL methods need to take the stress out of healthcare, for both caregivers and their patients. IDL methods need to incorporate the connectivity ecosystem available at and around the healthcare with patients detailing their issues and pain points over popular blogs as well as utilizing venues of the mobile virtual care to chat with their doctors, therapists and healthcare professionals from their smartphones or tablets. However, learning patient’s pain points covers wide range of diverse issues according to the different prospective of patients. This the reason that justifies conducting qualitative analytics (which focuses on detailed, individualized responses to open-ended questions) as opposed to quantitative analytics (which favors standardized questions and representative, statistically significant sample sizes). Figure 3 lists some of the popular patient pain points related to their journey in healthcare. It is important to notice that these patients’ pain points are highly subjective. Even if two patients have exactly the same problem, the underlying causes of that problem could differ greatly from one patient to another.

Too often to ease the impact of these pain points, it falls to physicians to provide solutions for their patients. While challenging, balancing evidence with patient perspectives is essential to protecting patient engagement and promoting more patient-appropriate medical decisions [15]. It will require physicians to improve their skills in compassion and expand their view of treatment beyond literature and guidelines to understand patient-specific, cultural, and societal contexts as well as to involve new skills to mine for further knowledge to provide prognostic views to their patients.

## III. Developing IDL Methods for Responding to Patient Pain Points Over the Internet

Advancements in technology including mobile and web applications have always had major impacts in medicine with tools and services. The smartphone and handheld devices are one of the most ubiquitous and dynamic trends in communication, in which one’s mobile phone can also be used for communicating via email, performing Internet searches, interacting with repositories including Electronic Health Records and using specific healthcare applications. The smartphone is one of the fastest growing sectors in the technology industry, and its impact in medicine has already been significant [16]. Social media channels such as Twitter are gaining increasing acceptance as mechanisms for instantaneous scientific dialogue with other caregivers as well as part of the physician’s medical practice [17], [18].

**FIGURE 4.**
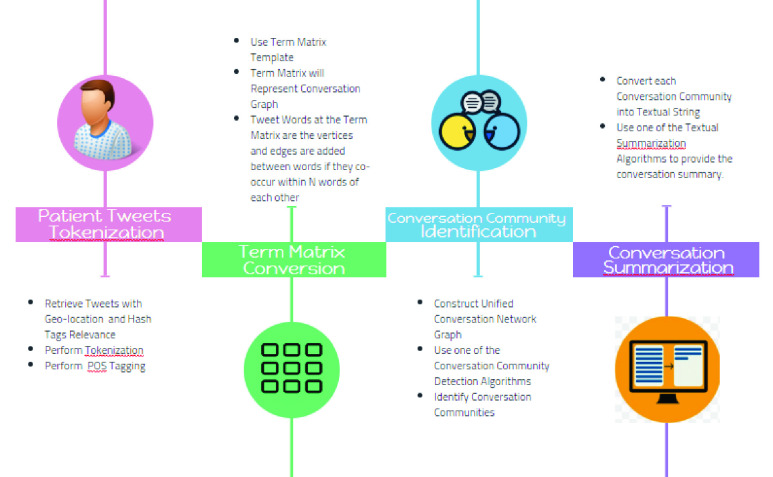
The twitter conversation crawler.

Our proposed exploratory IDL methods described in this section is based on the notion of Thick Data and it meant to be used by physicians and researchers for a specific or a narrow area of inquiry to explain everyday lives of patients and explores their pain points from social media like Twitter in order to understand patients set of preferences, attitudes, timelines, experiences, opinions, emotions, behavior, context, social dynamics and sensory information. We consider our proposed method as narrative inquiry that uses field texts such as tweets, conversations based on replies, retweets and physician generated hashtags, and conversational communities as the units of analysis to research and understand the way patients create meaning in their lives. Making sense of a life involves exploring the complex interactions between the caregiver and the conversational communities of patients and mediated by the particular topic, hashtag or features in patient lives over time. Thus meaning of a conversation is continually constructed and reconstructed. This construction and reconstruction occurs within, and is made visible through patient stories as expressed by their tweets. Stories reflect experience and can be crawled through the narratives. The conversations crawler is the first method in our proposed IDL set of methods (see Figure 4). The crawler starts by reading the tweets stream relevant to the physician geolocation or the hashtag or hashtags of this physician and convert the word sequence into a term matrix in which the rows are tweets and the columns are words. The values matching a document with a word in the matrix is counted using the *tf-idf* algorithm. This matrix is then used by a community graph construction algorithm like the *TextRank* [19] to produced highly focused graph of tweets that are related to certain search topic and context through adjusting the weights and context of the conversation lenses. Notable algorithms will be considered for the purpose of producing the primitive conversation graph including *Edge Betweenness, Walktrap, Label Propagations, SpinGlass and Louvain* [20]. To measure the robustness of the community detection algorithms to give insight in the strength of the divisions of a network into communities, we proposing to use the LFR measures and benchmarks [21] as well as a large dataset of health related tweets from reliable sources like the one in [22]. Based on the LFR measures on can select the most effective algorithm for detecting conversation communities. The conversation crawler produces summaries for each of the community conversations as well as visualizing the overall community conversation graph. The summarization algorithm that can be used by the crawler is a simple extraction-based summarization [23].

**FIGURE 5.**
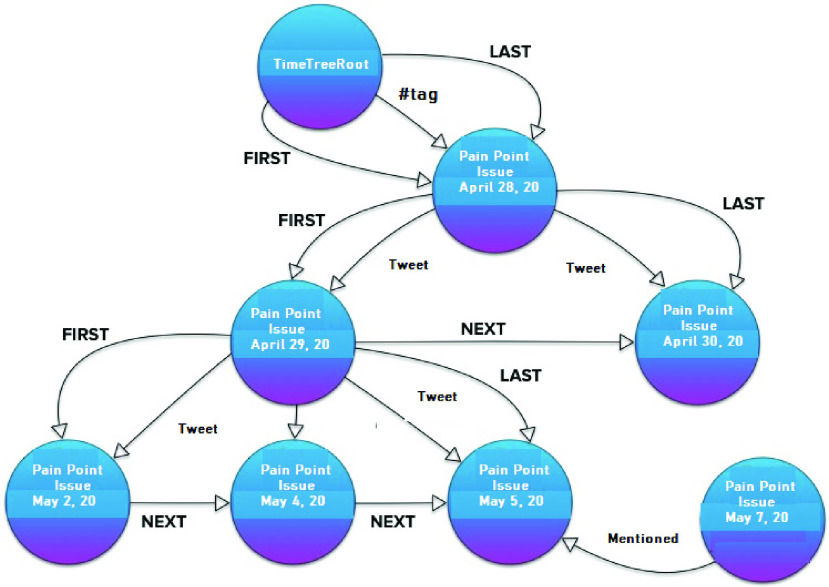
Conversation community timeline.

The first addition to the IDL methods is subdividing the conversational community networks based on the linkage style (e.g. retweet, mention and hashtag). Each style tweets conversation may be captured as a visualized story using the Neo4j.^1^ The timeline of these conversations about a pain point (e.g. like using #tag) can be displayed with Neo4j TimeTree API.^2^ Figure 5 illustrates the way such timeline visualization will be.^1^https://neo4j.com/^2^https://github.com/graphaware/neo4j-timetree

The following method will provide explorations for anomalies that can be detected from conversational communities. To tackle anomaly or abnormality detection problem, we are proposing to use many techniques have been developed in the past decades, especially for spotting outliers and anomalies in unstructured collections of multi-dimensional data points like those provided by the conversational communities. Anomaly detection in conversation networks refers to detecting users’ abnormal opinions and sentiment patterns as well as special temporal aspects of such patterns [24]. Since we have stored our conversational community’s networks in Neo4j graph database which offer huge advantages over traditional SQL or relational databases. Queries to detect anomalies can be expressed using clear Cypher^3^^3^https://neo4j.com/developer/cypher-query-language/ queries. For example, we know that fake accounts botnets over Twitter tend to share content in short bursts, using recently registered accounts with few connections [25]. To discover such anomaly we can run a simple Cypher query like this: that:

MATCH (account:Account)–(ip:IP)–(post:Post)

–(article:Tweet)

WHERE account.friends < 10 AND article.url =

‘http://wwww.GoDaddy.com’

AND post.timestamp >1588291200

AND post.timestamp < 1590969599

RETURN account

Which will return all Twitter accounts that have less than 10 follower’s connections and shared a link to http://wwww.GoDaddy.com between Friday, May 1, 2020 12:00:00 AM and Sunday, May 31, 2020 11:59:59 PM? However, results of the Cypher queries can be further visualized as a graph in Neo4j Keylines^4^^4^https://cambridge-intelligence.com/keylines/ API. Moreover, it quite common to use Cypher to query the conversation networks about routine thick data questions that have a good impact on the caregiver practice like:

Common Query 1: **Graph some of the caregiver mentions?**
MATCH
(u:Me:User)-[p:POSTS]->(t:Tweet)-[:MENTIONS]
->(m:User)
WITH
u,p,t,m, COUNT(m.screen_name) AS count
ORDER BY
count DESC
RETURN
u,p,t,m
LIMIT 10

Common Query 2: **List the most influential followers?**
MATCH
(follower:User)-[:FOLLOWS]->(u:User:Me)
RETURN
follower.screen_name AS user, follower.followers
AS followers
ORDER BY
followers DESC

Common Query 3: **The hashtags caregiver have used most often?**
MATCH
(h:Hashtag)<-[:TAGS]-(t:Tweet)
<-[:POSTS]-(u:User:Me)
WITH
h, COUNT(h) AS Hashtags
ORDER BY
Hashtags DESC
LIMIT 10
RETURN
h.name, Hashtags

Common Query 4: **What is my Follow back rate?**
MATCH
(me:User:Me)-[:FOLLOWS]->(f)
WITH
me, f, size((f)-[:FOLLOWS]->(me)) as
doesFollowBack
RETURN
SUM(doesFollowBack) / toFloat(COUNT(f)) AS
followBackRate

Common Query 5: **What are my Follower Recommendations - tweeting about me, but I don’t follow?**
MATCH
(ou:User)-[:POSTS]->(t:Tweet)-[mt:MENTIONS]
->(me:User:Me)
WITH
DISTINCT ou, me WHERE
(ou)-[:FOLLOWS]->(me)
AND NOT
(me)-[:FOLLOWS]->(ou)
RETURN
ou.screen_name

Common Query 6: **Provide links from interesting retweets?**
MATCH
(:User:Me)-[:POSTS]->
(t:Tweet)-[:RETWEETS]->(rt)-[:CONTAINS]
->(link:Link)
RETURN
t.id_str AS tweet, link.url
AS url, rt.favorites AS favorites
ORDER BY
favorites DESC
LIMIT 10

Common Query 7: **Who is tweeting with my top hashtags?**
MATCH
(me:User:Me)-[:POSTS]->(tweet:Tweet)-[:TAGS]
->(ht)
MATCH
(ht)<-[:TAGS]-(tweet2:Tweet)<-[:POSTS]
-(sugg:User)
WHERE
sugg <> me
AND NOT
(tweet2)-[:RETWEETS]->(tweet)
WITH
sugg, collect(distinct(ht)) as tags
RETURN
sugg.screen_name as friend, size(tags) as common
ORDER BY
Common DESC
LIMIT 20

**FIGURE 6.**
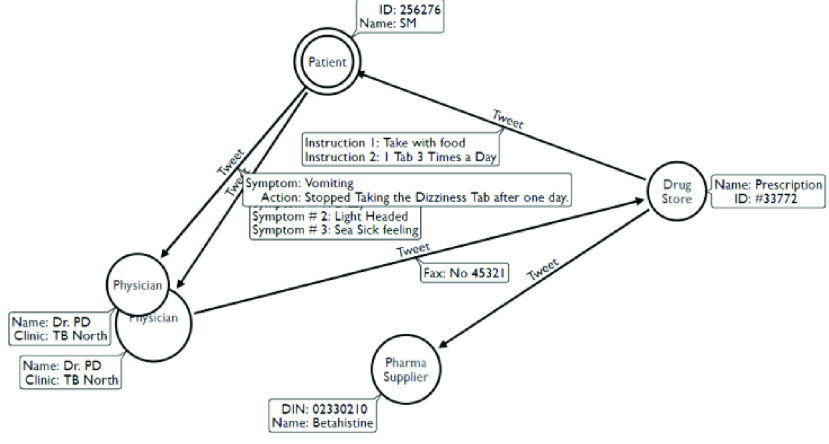
Conversation network revealing medication adherence problem.

If a physician wanted to create other type of queries then s/he can use the Arrows^5^^5^http://www.apcjones.com/arrows/# graphics editor to draw it and the editor will convert it into Cypher code. The next IDL method in our thick data analytics focuses on providing more context oriented graphs from the conversational communities like adherence to medical instructions graphs and compliance of providing a medical service. For example by in response to Common Query 6 we may end with a conversation network (Figure 6) that reveals medication adherence problem.

The last IDL method of this thick data framework is to use machine learning to infer the prognosis status of the patient cases from the conversation networks. This is a challenging area of research as machine learning is rarely been used on graph-based data like our conversation networks [26]. Machine learning on conversation networks can help us to do something like the followings:

• Predicting if a relationship exists between two nodes
• Scoring and classification of nodes, edges and whole graphs

Our machine learning IDL method will use node embedding (implemented using random walks) for link prediction. Embedding’s are often generated such that nearby nodes in the graph have similar embedding tensors [27]. Therefore a comparison between (e.g. Euclidean distances) provides a likelihood of linkage. Some methods like Node2Vec [28] actually directly train the embedding models on the presence/absence of links.

**FIGURE 7.**
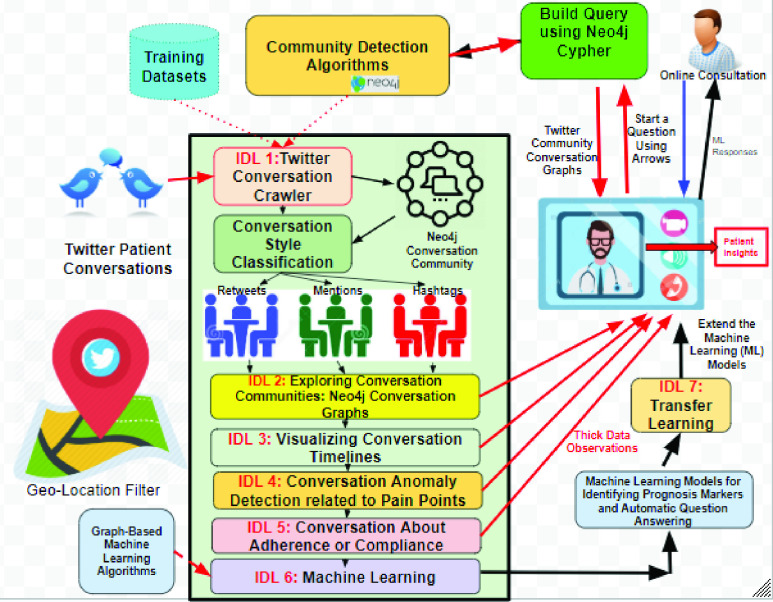
Overall thick data insight driven learning methods.

Our final step is to fine tune the conversation modeling process captured in the machine learning IDL method when new wave of tweets arrives with using transfer learning. Transfer learning has gained increasing attention due to the inferior performance of machine learning algorithms with insufficient training data. Most of the previous transfer learning methods and APIs (e.g. *ULMFiT, OpenAI GPT, ELMo, Stanford GloVe and Google AI’s BERT*), aim to learn a mapping function between feature spaces based on the inherent correspondence across the source and target domains or labeled instances. However, in many real world applications like our conversational networks, existing methods may not be robust when the correspondence across domains is noisy or labeled instances are not representative. We are experimenting recently with adopting a recent transfer learning via “Feature Isomorphism Discovery (TLFid)” [29] to discover common substructures across feature spaces and learning a feature mapping function from the target domain to the source domain and form and restructure the conversational networks on what currently called *community embedding* transfer learning [30]. To learn such embedding, our insight depends on the community detection algorithms and the node embedding techniques on the new arriving conversations. Node embedding will be used to identify new conversation communities to be used for community embedding [31]. Figure 7 illustrates our overall vision framework for learning patient insights from healthcare Twitter conversational networks.

## IV. Conclusion

Many healthcare organizations today are changing their systems to the new Value-Based Healthcare (VBHC) which is becoming a leading approach to improving patient and health system outcomes around the world [32]. It has been considered as the only way of organizing healthcare to transform health outcomes. However, the current healthcare systems does not allow to see the full interconnected parts contributing to the health outcomes. Few are taking a long-term holistic approach (e.g. focus groups, surveys, and scrapping data from healthcare websites) to gain insights and enhance outcomes. Much of these approached have high costs are driven by the lack of connected information and poor patient’s engagement. This article focuses on social media, in particular Twitter, as a source for patient insights due to its omnipresence, dynamism, engaging and geolocation focus. Twitter has enabled patient’s discussions to spread and evolve, adding a new channel of valuable insights for healthcare. With millions of discussions happening across Twitter networks in real time, the potential of listening to your patients on Twitter cannot be understated. This paper described a holistic vision to identify useful patient insights over Twitter relevant to the patient pain points using qualitative insights driven methods (IDLs). This visionary approach described five notable IDLs methods (IDL 1 to IDL 5) that can provide physicians and healthcare providers with meaningful insights about their local patient’s communities and their conversations. These methods start with identifying the conversation networks based on popular community detection algorithms and uses the graph algorithms provided by the Neo4j API to infer and visualize the relevant and focused conversations providing thick data observations to the physicians/healthcare givers who presented their questions on the values of their care as seen by the Twitter community around them. Moreover, this paper envisioned other two IDL methods to build machine learning models (IDL 6) for the inferred thick data observations based on node2vector techniques as well as to update these machine learning models whenever new conversation was detected using graph based transfer learning (IDL 7) using community embedding technique. The author is currently experimenting with these IDL methods using geo-located Twitter data relevant to the North Western Ontario region about patient tweets related to COVID-19 care. The source of the COVID-19 Twitter Data is from IEEE recent repository.^6^ More on these results are in the publication pipeline that is coming soon.^6^https://ieee-dataport.org/open-access/corona-virus-covid-19-geolocation-based-sentiment-data
